# Quantal Release of Dopamine and Action Potential Firing Detected in Midbrain Neurons by Multifunctional Diamond-Based Microarrays

**DOI:** 10.3389/fnins.2019.00288

**Published:** 2019-04-09

**Authors:** Giulia Tomagra, Federico Picollo, Alfio Battiato, Barbara Picconi, Silvia De Marchis, Alberto Pasquarelli, Paolo Olivero, Andrea Marcantoni, Paolo Calabresi, Emilio Carbone, Valentina Carabelli

**Affiliations:** ^1^Department of Drug and Science Technology and “NIS” Inter-departmental Centre, University of Torino, Turin, Italy; ^2^Department of Physics and “NIS” Inter-departmental Centre, University of Torino, Turin, Italy; ^3^Istituto Nazionale di Fisica Nucleare – Sezione di Torino, Turin, Italy; ^4^Experimental Neurophysiology Laboratory, IRCCS San Raffaele Pisana, University San Raffaele, Rome, Italy; ^5^University San Raffaele, Rome, Italy; ^6^Department of Life Sciences and Systems Biology and “NICO” Neuroscience Institute Cavalieri Ottolenghi, University of Torino, Turin, Italy; ^7^Institute of Electron Devices and Circuits, University of Ulm, Ulm, Germany; ^8^Neurological Clinic, Department of Medicine, Hospital Santa Maria della Misericordia, University of Perugia, Perugia, Italy

**Keywords:** diamond microelectrode arrays, midbrain neurons, quantal release, amperometric detection, spontaneous firing

## Abstract

Micro-Graphitic Single Crystal Diamond Multi Electrode Arrays (μG-SCD-MEAs) have so far been used as amperometric sensors to detect catecholamines from chromaffin cells and adrenal gland slices. Besides having time resolution and sensitivity that are comparable with carbon fiber electrodes, that represent the gold standard for amperometry, μG-SCD-MEAs also have the advantages of simultaneous multisite detection, high biocompatibility and implementation of amperometric/potentiometric protocols, aimed at monitoring exocytotic events and neuronal excitability. In order to adapt diamond technology to record neuronal activity, the μG-SCD-MEAs in this work have been interfaced with cultured midbrain neurons to detect electrical activity as well as quantal release of dopamine (DA). μG-SCD-MEAs are based on graphitic sensing electrodes that are embedded into the diamond matrix and are fabricated using MeV ion beam lithography. Two geometries have been adopted, with 4 × 4 and 8 × 8 microelectrodes (20 μm × 3.5 μm exposed area, 200 μm spacing). In the amperometric configuration, the 4 × 4 μG-SCD-MEAs resolved quantal exocytosis from midbrain dopaminergic neurons. KCl-stimulated DA release occurred as amperometric spikes of 15 pA amplitude and 0.5 ms half-width, at a mean frequency of 0.4 Hz. When used as potentiometric multiarrays, the 8 × 8 μG-SCD-MEAs detected the spontaneous firing activity of midbrain neurons. Extracellularly recorded action potentials (APs) had mean amplitude of ∼-50 μV and occurred at a mean firing frequency of 0.7 Hz in 67% of neurons, while the remaining fired at 6.8 Hz. Comparable findings were observed using conventional MEAs (0.9 and 6.4 Hz, respectively). To test the reliability of potentiometric recordings with μG-SCD-MEAs, the D_2_-autoreceptor modulation of firing was investigated by applying levodopa (L-DOPA, 20 μM), and comparing μG-SCD-MEAs, conventional MEAs and current-clamp recordings. In all cases, L-DOPA reduced the spontaneous spiking activity in most neurons by 70%, while the D_2_-antagonist sulpiride reversed this effect. Cell firing inhibition was generally associated with increased APs amplitude. A minority of neurons was either insensitive to, or potentiated by L-DOPA, suggesting that AP recordings originate from different midbrain neuronal subpopulations and reveal different modulatory pathways. Our data demonstrate, for the first time, that μG-SCD-MEAs are multi-functional biosensors suitable to resolve real-time DA release and AP firing in *in vitro* neuronal networks.

## Materials and Methods

### μG-SCD-MEA Fabrication

The employed substrates consisted of chemical vapor deposited single crystal diamonds produced by ElementSix^TM^ (Didcot, United Kingdom), with concentrations of substitutional nitrogen and boron impurities of 1 and 0.05 ppm, respectively. The crystals were cut along the (100) plane and the larger faces were polished.

The implantation process was performed at the AN2000 accelerator of the Laboratories of Legnaro of the Italian National Institute of Nuclear Physics (INFN), by employing a collimated 1.3 MeV He^+^ beam delivered on a 5 mm × 5 mm spot (Picollo et al., 2015a,b). The collimator was microfabricated by means of laser ablation of a 100 μm thick brass film (Kirana-laser, Rovereto, Italy). The implantation fluence (*F* = 1 × 10^17^ cm^-2^) was chosen to overcome the graphitization threshold (5 × 10^22^ vac cm^-3^–9 × 10^22^ vac cm^-3^) (Gippius et al., 1999; Khmelnitsky et al., 2015; Battiato et al., 2016) thus determining the full amorphization of a ∼250 nm thick and ∼2.3 μm deep layer below the sample surface. Subsequently, a thermal treatment at 950°C for 2 h in high vacuum (10^-6^ mbar) was performed in order to promote the complete graphitization of the modified regions (Hoffman et al., 1996; Hickey et al., 2009; Picollo et al., 2012). A preliminary electrical characterization of the device was then performed as previously described (Picollo et al., 2016a).

### Ion-Induced Damage Simulation

The ion-induced damage density profiles were obtained using a numerical simulation performed with the “Stopping and Range of Ion in Matter” SRIM-2013.00 Monte Carlo code (Ziegler et al., 2010) in “Detailed calculation with full damage cascades” mode, by setting a displacement energy value of 50 eV (Wu and Fahy, 1994). The output of the simulation process (i.e., the number of vacancies created per unit of depth and ion, in #vac #ions^-1^ μm^-1^ units) was then multiplied by the ion fluence, assuming the absence of non-linear effects such as damage saturation or formation of defect complexes.

### Cell Cultures

The methods for the primary culture of mesencephalic dopamine neurons from substantia nigra (SN) was adapted from Pruszak et al. (2009). The ventral mesencephalon area was dissected from embryonic (E15) C57BL6 TH-GFP mice (Sawamoto et al., 2001; Matsushita et al., 2002). TH–GFP mice were kept heterozygous via breeding TH–GFP mice with C57BL/6J mice. All animals were housed under a 12-h light/dark cycle in an environmentally controlled room with food and water ad libitum. All experiments were conducted in accordance with the European Community’s Council Directive 2010/63/UE and approved by the Italian Ministry of Health and the Local Organism responsible for animal welfare at the University of Turin (Authorization DGSAF 0011710-P-26/07/2017).

HBSS (Hank’s balanced salt solution, without CaCl_2_ and MgCl_2_), enriched with 0.18% glucose, 1% BSA, 60% papain (Wortington, Lakewood, NJ, United States), 20% Dnase (Sigma-Aldrich) was stored at 4°C and used as digestion buffer. Neurons were plated at final densities of 600 cells mm^-2^ on petri dishes, or 2000 cells mm^-2^ on conventional MEAs or μG-SCD-MEAs. Cultured neurons were used at 7 DIV for current-clamp experiments and at 14 DIV with MEAs or μG-SCD-MEAs. Petri dishes, as well as MEAs and μG-SCD-MEAs were coated with poly-L-Lysine (0.1 mg ml^-1^) as substrate adhesion. Cells were incubated at 37°C in a 5% CO_2_ atmosphere, with Neurobasal Medium containing 1% pen-strep, 1% ultra-glutamine, 2% B-27 and 2.5% FBSd; pH 7.4.

### Solutions

For current-clamp experiments the intracellular solution contained (mM): 135 gluconic acid (potassium salt: K-gluconate), 5 NaCl, 2 MgCl_2_, 10 HEPES, 0.5 EGTA, 2 ATP-Tris and 0.4 Tris-GTP. The extracellular solution (Tyrode’s solution) contained (mM): 130 NaCl, 4 KCl, 2 CaCl_2_, 2 MgCl_2_, 10 glucose and 10 HEPES; pH 7.4.

For amperometric recordings, exocytosis was stimulated by means of a KCl-enriched solution, containing (mM): 100 NaCl, 2 MgCl_2_, 10 glucose, 10 HEPES, 10 CaCl_2,_ and 30 KCl (pH 7.4).

Potentiometric recordings with MEAs and μG-SCD-MEAs were performed while keeping the cells under a controlled CO_2_-enriched atmosphere and stable temperature conditions.

### Amperometric Recordings

Amperometric recordings were performed by means of μG-SCD-MEAs (4 × 4 channels geometry) and dedicated electronics, which were designed at the Institute of Electron Devices and Circuits (Ulm University). The whole electronic chain was inserted into a Faraday cage to minimize noise. The chip carrier was directly plugged-in to the front-end electronics connected to a data acquisition unit (National Instruments USB-6216). The circuit was grounded by means of a reference Ag/AgCl electrode, which was immersed in the extracellular solution. Amperometry was performed by holding the 16 electrodes at a constant potential of +0.65 V relative to the Ag-AgCl reference electrode.

The acquisition electronics consisted of low-noise transimpedance amplifiers with an input bias current of 1 pA and a gain, set by feedback-resistors, of 100 MΩ. The amplified signals were filtered at 4 kHz with 4^th^ order Bessel low-pass filters and were subsequently acquired at a sampling rate of 25 kHz per channel. The National Instruments DAQ interface was connected to a computer via a high-speed USB. We used data acquisition control software that was developed in LabView. The noise level was evaluated in spike-free trace segments and then averaged over the 16 electrodes, leading to a mean amplitude of 5.5 ± 0.7 pA, with a mean signal-to-noise ratio (S/N) of ∼3. Spike analysis was performed using “Quanta Analysis” routine (Mosharov and Sulzer, 2005) in Igor pro 5.00 data analysis software by waveMetrics. No change in current output was observed when electrode polarization was lowered below 50 mV.

### Potentiometric Recordings Using μG-SCD-MEAs and MEAs

Potentiometric recordings were performed while the cells were kept in their culture medium. Recordings took place inside a dedicated incubator, at a controlled temperature and 5% CO_2_ atmosphere. A MCS MEA 1060-Inv-BC amplifier from Multi Channel Systems (Reutlingen, Germany) was used as the read-out unit for both with μG-SCD-MEAs (8 × 8 geometry) and conventional MEAs (60MEA200/300iR-Ti).

Data acquisition was controlled using MC_Rack software. The threshold for spike detection was set at -30 μV and the sampling frequency at 10 kHz. Data were then analyzed using Clampfit software (Molecular Devices, Silicon Valley, CA, United States).

### Current Clamp Recordings

Patch-clamp experiments were performed using Pclamp software (Molecular Devices, Silicon Valley, CA, United States). All experiments were performed at a temperature of 22–24°C. Data analysis was performed using Clampfit software.

### Fluorescence Images

Images were acquired using a Zeiss microscope primovert 40x (Carl Zeiss, LLC United States). In the fluorescence configuration, samples were excited with radiation in the visible spectrum at a characteristic wavelength λ_ex_ = 470 nm, and the emission wavelength was λ_em_ = 505 nm, which is typical of GFP staining.

### Statistical Analysis

Data are given as mean ± SEM, for n number of cells, except where otherwise specified. Statistical significance was estimated using unpaired Student’s *t*-tests. Data were considered statistically significant when *p* ≤ 0.05. Statistical analysis was performed with Origin 8.5 software (OriginLab Corporation, Northampton, MA, United States).

## Introduction

Dopamine (DA) plays fundamental roles in a variety of neurophysiological functions and neurological diseases. Dopaminergic microcircuits are involved in movement, reward, memory and cognition (Waelti et al., 2001), while the degeneration of the nigrostriatal pathway in Parkinson’s disease (PD) impairs control and planning of movement, causing tremors and postural instability.

Fluctuations of DA concentration occur on the seconds to subseconds time scale, thus making them suitable for study with carbon fiber electrodes (CFEs) and fast-scan cyclic voltammetry (Hafizi et al., 1990; Kawagoe and Wightman, 1994; Heien and Wightman, 2006; Patel and Rice, 2013). However, *in vivo* DA electrochemical detection is hampered by electrode fouling, which is caused by accumulation of oxidized products and by interference of ascorbic and uric acid, ultimately limiting electrode sensitivity and selectivity (Suzuki et al., 2007).

Since the early investigation of synaptic dysfunction is a target when attempting to understand the molecular mechanisms that lead to neurodegenerative processes, the development of mutifunctional sensing tools for simultaneous monitoring neurotransmitter release and electrical activity are extremely relevant for addressing key aspects of neurotransmission in the early stages of neurodegenerative diseases (Suzuki et al., 2013; Schirinzi et al., 2016; Castagnola et al., 2018; Ghiglieri et al., 2018; Picconi et al., 2018).

In this regard, conventional multielectrode arrays (MEAs) have been employed to investigate the firing properties in SN pars compacta slices (Berretta et al., 2010), while amperometric detection of DA release from cultured neurons, was initially performed using CFEs (Pothos et al., 1998; Pothos, 2002; Staal et al., 2004; Mosharov and Sulzer, 2005). A range of different amperometric microarrays to detect DA release from PC12 cells (Chen et al., 1994; Lin et al., 2012; Trouillon and Ewing, 2014), striatal slices (Suzuki et al., 2013) and from isolated dopaminergic somas from the pond snail *Lymnaea stagnalis* have been designed (Patel et al., 2013). Dopamine release from striatal slices has been detected by carbon nanotube multi electrode arrays and the same device could successfully detect APs and field post-synaptic potentials from cultured hippocampal neurons and slices (Suzuki et al., 2013). In spite of this, no data concerning the detection of quantal release and electrical activity from the same cultured neurons using the same multiarray prototypes have been reported, to the best of our knowledge. Micro-graphitic single-crystal diamond multielectrode arrays (μG-SCD-MEAs) are a powerful sensor for investigating neurosecretion in living cells (Picollo et al., 2013, 2015b). Previous findings have demonstrated their ability to monitor spontaneous and evoked quantal catecholamine release from cultured mouse and bovine adrenal chromaffin cells (Picollo et al., 2016b) as well as from fresh mouse adrenal slices (Picollo et al., 2016a; Carabelli et al., 2017). Besides providing simultaneous recordings from a variety cells, which have been plated and cultured on the planar array for a number of days, μG-SCD-MEAs possess high-time resolution and sensitivity for the detection of amperometric events with different shape, such as the small amplitude, or previously identified “stand-alone-foot” events (Picollo et al., 2016a). Taking advantage of diamond biocompatibility (Bonnauron et al., 2008; Nistor et al., 2015; Piret et al., 2015; Alcaide et al., 2016), we have succeeded in culturing primary midbrain neurons on μG-SCD-MEAs. In the present work, we have provided the first evidence that μG-SCD-MEAs can detect the quantal exocytosis of neuronal synaptic vesicles as well as spontaneous neuronal firing activity.

## Results

### Fabrication of μG-SCD-MEAs

The electrochemical sensors that have been used in the present work consist of multi electrode arrays with either 16 or 60 graphitic electrodes that have been embedded into an artificial single-crystal diamond substrate. The two devices combine the properties of diamond, including: (1) biocompatibility, guaranteeing the plating and maintenance of primary cultures for weeks (Tang et al., 1995; Nistor et al., 2015); (2) chemical inertness, which prevents modifications to the employed solutions; and (3) wide optical transparency.

**FIGURE 1:**
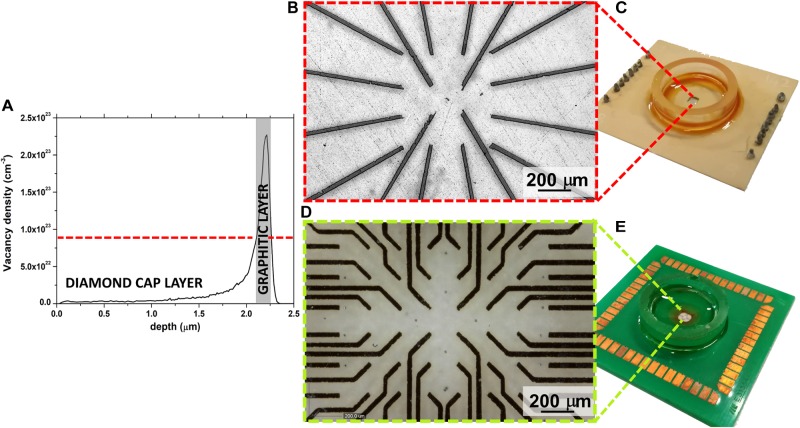
**(A)** Monte Carlo Simulation of vacancy density profile induced in diamond by 1.3 MeV He^+^ ions; a gray rectangle highlights the graphitized region. **(B)** Optical micrograph of the 16 channels μG-SCD-MEA with magnification of the graphitic electrodes in the central region of the sensor **(C)**; **(D)** optical micrograph of the 60 channels μG-SCD-MEA with the magnification of the graphitic electrodes in the central region of the sensor **(E)**.

Conventional fabrication schemes cannot be used for the assembly of these sensors due to the extreme chemical/physical characteristics of diamond, meaning that an advanced MeV-ion-beam-implantation-based process was used (Olivero et al., 2010; Picollo et al., 2015b). This fabrication technique allows the selective phase transition from the diamond to graphite to be promoted by taking advantage of the metastable nature of the substrate. Indeed, if the density of ion beam-induced defects (commonly parametrized in terms of vacancy density) overcomes a critical threshold, the graphitization of the damaged region is obtained upon high-temperature thermal treatment. Moreover, the fact that the defects created by irradiation with MeV ions follow a typical distribution, which is characterized by the so-called “Bragg peak” and the ion end of range (Figure 1A), means the position of the graphitic electrodes can be modulated along the substrate depth, thus guaranteeing intrinsic electrical passivation due to the presence of the diamond cap layer. Only the electrodes end-points emerge to the surface, thus allowing the interfacing with the front-end electronic in the peripheral region of the sensor and the cells coupling in the central region.

Figure 1B,C shows the sensor and the magnification of the graphitic electrodes, which were arranged in a 4 × 4 matrix, while Figure 1D,E show analogous representations for the device with a 8 × 8 matrix of electrodes (without the four electrodes on the corners). In both cases, active areas were regularly spaced with a ∼200 μm step.

### Real-Time Detection of Quantal DA Release From Midbrain Neurons Using μG-SCD-MEAs

**FIGURE 2:**
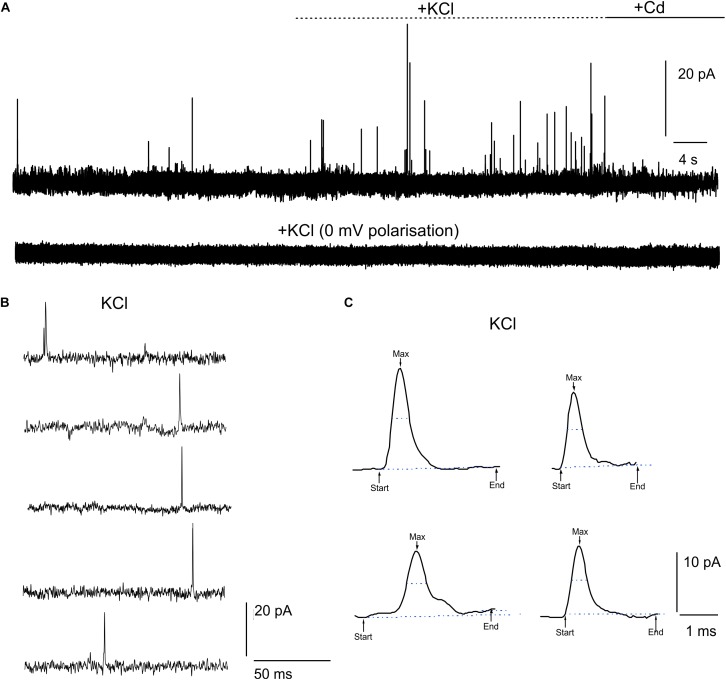
Detection of quantal dopamine release by μG-SCD-MEAs. **(A)** Representative recordings from one channel of μG-SCD-MEAs (4 × 4). DA release occurred spontaneously in 2 mM CaCl_2_; following stimulation with 30 mM KCl, the frequency of release increased without significantly altering spike parameters (see text for details). No events can be detected when the recording electrodes were polarized at 0 mV (bottom trace). **(B)** Example of multi-site detection of DA release from five different graphitic electrodes. **(C)** Examples of different amperometric spikes shown in expanded time scale.

DA release from cultured midbrain neurons can either occur at the somato-dendritic or the axon-terminal level (Rice and Patel, 2015). In this work, we have cultured midbrain neurons for 20 DIV on μG-SCD-MEAs and found that quantal exocytotic events can be detected after 10 DIV. Under our experimental conditions, the density of cell plating on the multiarray (see section “Materials and Methods”), allowed us to reveal amperometric signals from approximately 25–30% of the electrodes. Unstimulated (i.e., spontaneous) release was barely detectable (5% of trials) and occurred at low frequency (0.11 ± 0.07 Hz) in 2 mM CaCl_2_ (Figure 2A). Amperometric spikes were characterized by a mean maximum current amplitude (I_max_) of 13.2 ± 1.0 pA and a half-time width (t_half_) of 0.57 ± 0.03 ms (*n* = 5). Stimulation with 30 mM KCl (Figure 2A–C) increased the release frequency to 0.40 ± 0.03 Hz. However, spike parameters were unaffected: I_max_ was 18.5 ± 1.1 pA and t_half_ was 0.52 ± 0.01 ms (*n* = 13 cells, from 4 μG-SCD-MEAs). On the other hand, 200 μM CdCl_2_ suppressed Ca^2+^-dependent exocytosis through voltage-gated Ca^2+^ channels, as shown in Figure 2A. No events were detected when the recording electrodes were polarized to 0 mV to nullify dopamine detection (bottom trace). Representative recordings of simultaneous acquisition from five different electrodes of the same μG-SCD-MEA are shown in Figure 2B: multiple events, such as the one visible in the first trace, were discarded from the analysis. Some representative spikes that were recorded in the presence of KCl are shown at a more expanded time scale in Figure 2C.

### Detection of Spontaneous AP Firing From Cultured Midbrain Neurons

After assessing the sensitivity of μG-SCD-MEAs to reveal quantal DA release in dopaminergic neurons, we tested if these sensors were able to measure the electrical activity of cultured midbrain neurons. For this purpose, μG-SCD-MEAs were patterned with a higher electrode number (8 × 8 array) than to those used for amperometry (4 × 4 array). Recordings were performed in parallel using μG-SCD-MEAs and conventional MEAs [Multi Channel Systems (MCS)], for a more rigorous interpretation of acquired data. As has already been observed in cultured hippocampal neurons (Gavello et al., 2012, 2018; Allio et al., 2015) and other brain regions (Martinoia et al., 2005), mesencephalic DA neurons start generating spontaneous APs after 7 DIV (Henderson et al., 2016), while network functionality was well-resolved at 14 DIV.

**FIGURE 3:**
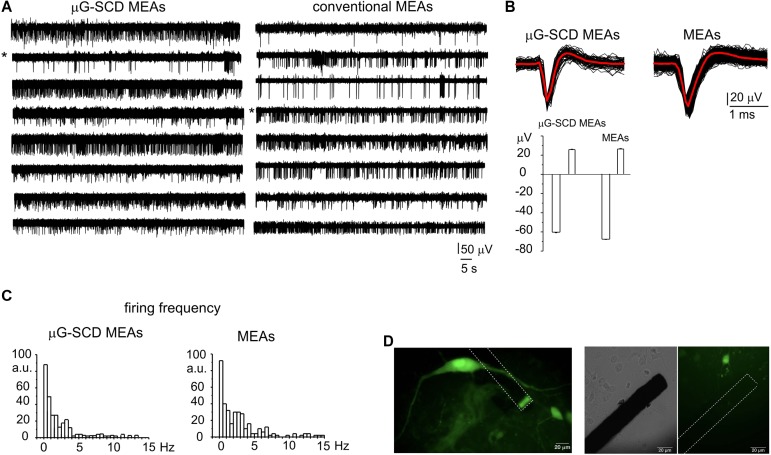
Potentiometric recordings from μG-SCD-MEAs and MEAs. **(A)** Representative spontaneous firing activity of cultured midbrain neurons detected using μG-SCD-MEAs (left) and MEAs (right). **(B)** Top: representative extracellularly detected APs shown on an expanded time scale for both devices. The red trace is the averaged signal. Bottom: the bar histogram represents the mean peak amplitude (negative deflection) and antipeak amplitude (positive deflection) of the extracellularly recorded APs. Data refer to the traces indicated by the asterisks in **(A). (C)** Firing frequency distribution for μG-SCD-MEAs and MEAs. Number of data are reported as percentages, indicated as arbitrary units (a.u.). **(D)** GFP-TH positive staining neurons cultured on μG-SCD-MEA (left). Dashed lines represent the buried graphitic channels inside the diamond bulk. The graphitic emerging (sensing) electrode appears as rectangular shaped. Middle, right: a single graphitic channel on an expanded scale in the proximity of a dopaminergic neuron. The same image is shown in transmitted light (middle) and in fluorescence (right).

Representative recordings of spontaneously firing midbrain neurons, measured using μG-SCD-MEAs and conventional MEAs, are shown in Figure 3A. This spontaneous spiking activity occurred under physiological conditions (2 mM Ca^2+^) and was suppressed by blocking the firing during the exogenous application of 300 nM TTX (data not shown). Unlike amperometric spikes, which exibit monopolar waveforms, single APs (Figure 3B) were characterized by a fast downward deflection (negative peak), which corresponds to the AP rising phase, followed by an upward deflection (positive antipeak), which is associated to the AP repolarising phase (Fromherz, 1999; Marcantoni et al., 2007). The mean amplitude of the negative peaks recorded by μG-SCD-MEAs (*n* = 10) was -50.2 ± 3.6 μV, with S/N of ∼4, while the mean signal amplitude was equal to -54.0 ± 4.7 μV, with S/N of ∼5, for conventional MEAs (*n* = 10). The amplitude of the positive antipeak, when detectable, was approximately 30% of the negative peak amplitude. For instance, for the channels indicated by the asterisks in Figure 3A, the mean positive antipeak amplitudes was 25.4 ± 0.4 and 26.0 ± 0.4 μV (with μG-SCD-MEAs and MEAs, respectively), while the mean peak amplitude was -60.1 ± 0.6 and -67.1 ± 0.5 μV (with μG-SCD-MEAs and MEAs, respectively). Since the positive antipeak was not detectable in all neurons, we limited our analysis to the negative peak, in good agreement with our previous observations (Vandael et al., 2010).

Firing frequencies were comprised within 0.1 and 15 Hz (Figure 3C), which is consistent with the presence of distinct neuronal populations within the network (Berretta et al., 2010; Ramayya et al., 2014). Most neurons (67%) were spontaneously active, fired with a basal frequency which not exceeded 4 Hz and had a mean firing frequency of 0.66 ± 0.14 or 0.90 ± 0.10 Hz, when measured with μG-SCD-MEAs and conventional MEAs (*p* > 0.1), respectively. The remaining neurons had a much higher basal firing frequency, ranging between 4 and 11 Hz and characterized by mean values of 6.8 ± 1.4 and 6.4 ± 0.5 Hz, when measured with μG-SCD-MEAs and MEAs (*p* > 0.1), respectively. This heterogeneity of responses can, most likely, be ascribed to the presence of distinct neuronal populations such as DA neurons from SN, GABAergic and DA neurons from the nearby ventral tegmental area (Berretta et al., 2010; Cucchiaroni et al., 2011). Optical images of GFP-TH^+^ neurons that were cultured on μG-SCD-MEAs are provided in Figure 3D.

Despite the above-mentioned heterogeneity, these recordings are the first experimental evidence that μG-SCD-MEAs are suitable for potentiometric recordings from primary cultures of brain neurons.

### D_2_-Autoreceptor Induced Inhibition of Repetitive Firing in Current-Clamp Recordings

**FIGURE 4:**
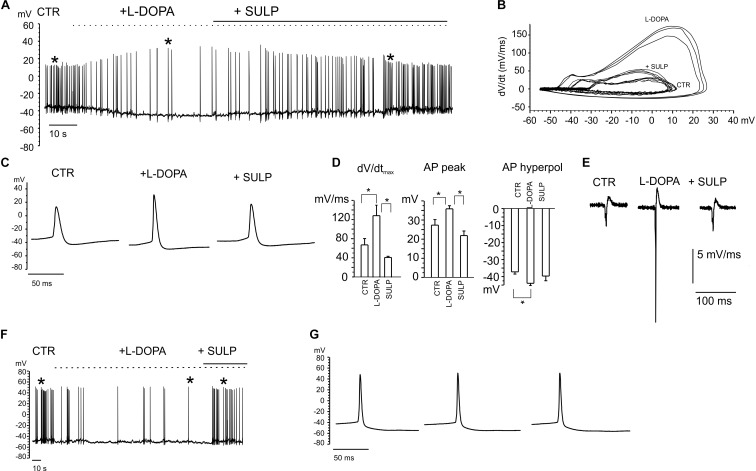
Current-clamp recordings exhibiting the features of L-DOPA induced modulation of neuronal firing. **(A)** Repetitive firing activity is inhibited by L-DOPA (20 μM) and restored by blocking the D_2_ autocrine receptors. Firing inhibition is accompanied by a membrane hyperpolarization and increased peak amplitude. **(B)** Phase-plane plot analysis of the action potential shown in **(A)**. **(C)** Signals identified by the asterisk in **(A)** are shown through an enlarge scale. **(D)** The following parameters have been calculated from the phase-plot analysis: maximum dV/dt versus V, action potential (AP) peak amplitude, AP hyperpolarization peak. Data are expressed as mean values. Statistical difference (^∗^*p* < 0.05) is indicated on the bar histogram. **(E)** Negative first derivative of signals reported in **(C)**. **(F)** Example of firing inhibited by L-DOPA without significant alterations in the AP peak amplitude, shown in **(G)** on an enlarged scale.

The firing of nigral dopaminergic neurons is down-regulated by DA release through a D_2_-autoreceptor mediated pathway (Aghajanian and Bunney, 1977; Mercuri et al., 1990). Since this down-regulatory pathway has been observed in midbrain slices and we were recording from primary cultured midbrain dissociated neurons (Lacey et al., 1987; Guatteo et al., 2013), we aimed to identify this inhibitory down-regulation under our experimental conditions first (Bigornia et al., 1990). Preliminary experiments were performed in whole-cell *current-clamp* configuration, by applying the DA precursor levodopa (L-DOPA) (20 mM). Recordings were selectively performed on 7 DIV dopaminergic neurons that were identified by means of GFP staining (Figure 3D). Although the responses to applied L-DOPA varied, it caused a 70 ± 4% reduction of the firing frequency in 80% of cases (*n* = 20 cells, from 1.36 ± 0.02 to 0.41 ± 0.11 Hz; Figure 4A,F). Maximum inhibition occurred within 2–5 min of L-DOPA perfusion, and was reversed some minutes after the application of the D_2_ antagonist sulpiride (10 μM). The repetitive firing frequency measured in the presence of the D_2_ antagonist recovered to 1.2 ± 0.2 Hz, thus confirming the autocrine inhibition that is induced by released DA (Guatteo et al., 2013). It is worth mentioning that the reduced firing frequency was associated, in 70% of the cases, to a membrane hyperpolarization of -7.8 ± 1.1 mV and by a sharp increase in AP peak amplitude (from 27 ± 3 to 35.6 ± 1.6 mV; *n* = 14, *p* < 0.05; Figure 4C,D). All this was most likely induced by the DA-mediated activation of a G-protein-coupled potassium channel (GIRK) (Lacey et al., 1987). Both effects were reversed after perfusion with sulpiride.

In the remaining 30% of neurons, the nearly threefold reduction of firing frequency occurred without causing either the significant hyperpolarization of the membrane potential, or alterations in the AP waveform (Figure 4F,G). For this subset of neurons, in some cases sulpiride restored the control firing frequency, even though the recovery was not always complete. This variability reveals the probable existence of distinct modulatory pathways that may originate from different midbrain neuron subpopulations (Dragicevic et al., 2015; Duda et al., 2016).

Phase plane plot analysis was performed in order to gain further insights into the APs properties and their modulation by L-DOPA (Vandael et al., 2012; Marcantoni et al., 2014). By plotting the time derivative of voltage versus voltage (*dV/dt*), parameters such as the AP threshold can be easily inferred from the voltage value at which dV/dt suddenly increases. The phase-plane plots in Figure 4B are referred to the same APs that are indicated by the asterisks in Figure 4A. From the plot we found that: (i) the maximum derivative (dV/dt_max_), which is associated with the maximum current density through voltage-gated Na_v_ channels, was drastically enhanced by L-DOPA (from 67 to 129 mV ms^-1^, *p* < 0.05, Figure 4D), suggesting a sustained recruitment of Na_v_ channels (Guarina et al., 2018); (ii) the AP hyperpolarization peak was significantly augmented by L-DOPA, from from -37.2 ± 1.3 to -40 ± 2 mV (*p* < 0.05, Figure 4D); (iii) the AP threshold, measured from the phase-plane plot when an abrupt change in dV/dt was observed (at 4.5 ± 1.2 mV ms^-1^ for control and 6.4 ± 0.9 mV ms^-1^ for L-DOPA-treated neurons), decreased from -25.3 ± 1.8 to -31.9 ± 1.8 mV (*p* < 0.05), respectively. This again confirms a potentiated recruitment of Na_v_ channels during L-DOPA treatment.

In order to compare the AP waveform recorded intracellularly with those recorded extracellularly, the negative first derivative of AP traces shown in Figure 4C is reported in Figure 4E. They correspond to the AP shape recorded extracellularly by the MEAs (Fromherz et al., 1991) and identified as a biphasic AP waveform in which, similarly to that of Figure 3B, a large negative peak and a small positive antipeak component can be distinguished.

Finally, a range of different effects on neuronal activity were detected in the neurons that were not inhibited by L-DOPA (20% of neurons). L-DOPA accelerated repetitive firing by 80 ± 20% (17% of neurons), while it was ineffective in the remaining ones (3% of neurons).

### Heterogeneity of L-DOPA Induced Responses in Cultured Midbrain Neurons Observed Through μG-SCD-MEAs and Conventional MEAs

**FIGURE 5:**
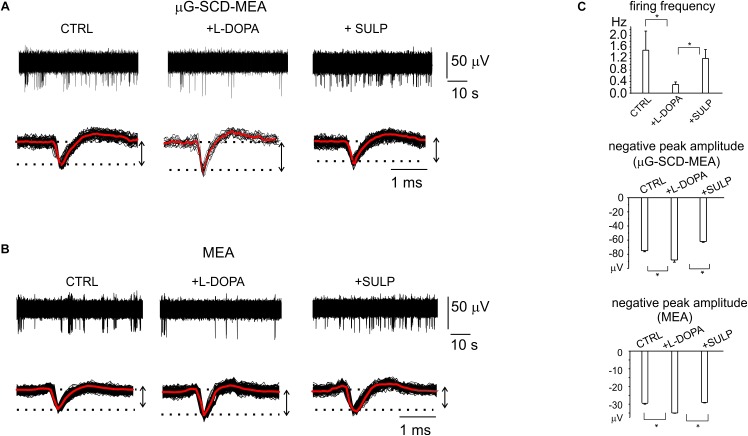
D2-autoreceptor mediated down-regulation of neuronal firing activity measured by μG-SCD-MEAs and MEAs. **(A)** Representative recordings from one channel of μG-SCD-MEA. Bottom: potentiometric signals from **(A)** are visualized on an enlarged scale (black traces). Averaged signals are shown in red. **(B)** Representative recording from one electrode belonging to a conventional MEA, showing the reduction of the firing frequency and its recovery induced by sulpiride. Bottom: individual (black) and averaged signals (red). **(C)** *Top*: the bar histograms represent the mean firing frequency in the three conditions (control, with L-DOPA and with L-DOPA+sulpiride), measured by conventional MEAs. *Centre, bottom*: the bar histogram shows the mean values of the negative peak amplitude and related statistical significance (^∗^*p* < 0.05), for μG-SCD-MEAs and MEAs, respectively.

Potentiometric recordings using μG-SCD-MEAs were performed to simultaneously detect spikes arising from different neuronal populations and to investigate their responses to the applied drugs. With respect to patch-clamp experiments, performed on isolated and young neurons (7 DIV), these trials were designed to provide a rapid screening of the effects of L-DOPA on mature networks (14 DIV). After the firing properties under control conditions were monitored for a couple of minutes, the addition of L-DOPA to the culture medium revealed three different responses, confirming the existence of heterogeneous firing, as measured in SN slices (Berretta et al., 2010). In most cases (70% of neurons), the firing activity was significantly reduced by L-DOPA and the inhibitory effect required some minutes for completion (Mercuri et al., 1990). As shown in a representative recording using μG-SCD-MEAs, the firing frequency was reduced by 80% after 2–3 min, and the extracellular AP peak increased from -75 ± 1 to -87 ± 3 μV, while sulpiride reversed both effects, suggesting that D_2_ autoreceptors are involved (Figure 5A,C). On average L-DOPA decreased the spontaneous spiking activity from 1.1 to 0.3 Hz and increased the negative peak amplitude by 14% (*n* = 5 μG-SCD-MEAs, *p* < 0.05), suggesting a prominent recruitment of Na_v_ channels following L-DOPA hyperpolarization. In order to validate these experimental findings, we repeated the same experiments using conventional MEAs. Once again, the majority of neurons (64%), responded to L-DOPA by reducing the mean firing frequency, on average from 1.5 ± 0.7 to 0.29 ± 0.09 Hz (*p* < 0.05, *n* = 10 MEAs; Figure 5B), while sulpiride restored the basal frequency to 1.1 ± 0.3 Hz (Figure 5C). In this subset of neurons, firing frequency reduction was also associated to a 20% increase in the negative peak amplitude, confirming the prominent role that D_2_-autoreceptors play in L-DOPA induced inhibition.

**FIGURE 6:**
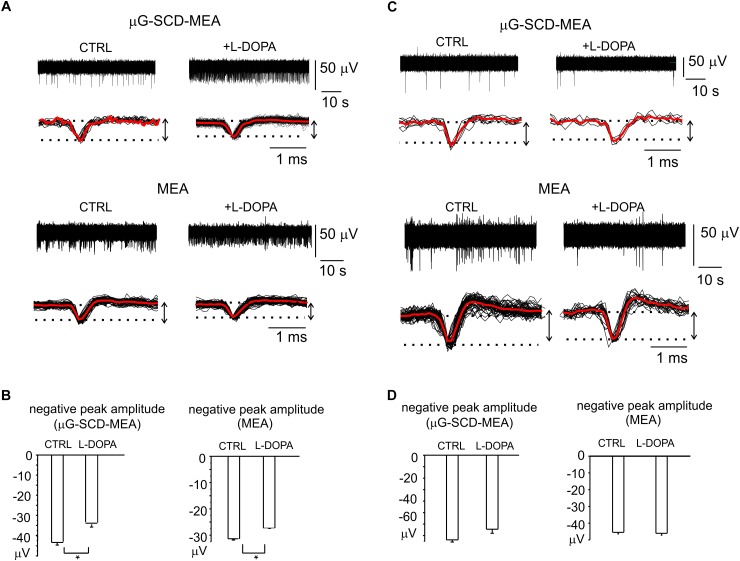
Heterogeneity of L-DOPA-induced responses on midbrain neurons viewed through μG-SCD-MEAs and MEAs. **(A)** Representative recordings of L-DOPA-induced potentiation of neuronal firing, respectively acquired by μG-SCD-MEAs (*top*) and MEAs (*bottom*). Under each recording, individual (black) and averaged signals (red) are shown on an expanded time scale. **(B)** L-DOPA induced potentiation is associated to the reduced amplitude of the AP peak, as shown in the bar histograms. **(C)** L-DOPA reduces the firing frequency without causing any significant alteration in the extracellularly recorded peak amplitude. Significant recordings detected by μG-SCD-MEAs (*top*) and MEAs (*bottom*). **(D)** The bar histograms show the mean negative peak amplitude, which is not affected by L-DOPA, for both devices.

Nevertheless, a relevant fraction of neurons in the mature networks (30 and 36%, respectively for μG-SCD-MEAs and MEAs) also displayed a significant increase (up to sixfold) in spontaneous frequency and a 30% reduction in the negative peak amplitude following exposure to L-DOPA. This is in good agreement with the heterogeneity of responses that we observed in dissociated neurons under current-clamp conditions. In the example shown in Figure 6A for μG-SCD-MEAs, the negative peak amplitude decreased from -44.1 ± 1.2 to -34.2 ± 1.1 μV, while the firing frequency increased from 0.5 to 2.9 Hz (Yasumoto et al., 2004). Similarly, the potentiation of firing activity by L-DOPA occurred with a mean threefold increase in firing frequency when using conventional MEAs, and was usually accompanied by a 28% decrease in the negative peak amplitude (Figure 6B).

L-DOPA reduced the spiking activity without affecting the AP shape in the remaining cases. Examples of this modulation are shown in Figure 6C. The unaltered amplitude of the negative AP peak, that have been revealed by μG-SCD-MEAs and conventional MEAs (Figure 6D), confirms the findings obtained under patch-clamp conditions, in which 30% of neurons displayed a reduced firing frequency without alterations of the AP rising phase.

## Discussion

We have provided the first evidence that μG-SCD-MEAs allow long-term neuronal cultures to be performed and that they can function as sensing devices for recording quantal exocytosis and spontaneous AP firing.

### Amperometric Detection of Quantal Dopamine Release

Amperometric recordings have proved that μG-SCD-MEAs are suitable for the real time detection of exocytosis from neuronal networks. Under physiological conditions (2 mM Ca^2+^), μG-SCD-MEAs can resolve spontaneous secretory events as amperometric spikes of <20 pA I_max_ and mean quantity of charge Q of 0.01 pC, which can most likely be associated with the tonic discharge activity of the network (Sulzer et al., 2016). These exocytotic events are significantly smaller than those of released catecholamines from large dense-core vesicles of adrenal chromaffin cells, whose I_max_ is in the order of 10s of pA, and Q is > 1.5 pC, as has already been reported using the same μG-SCD-MEAs (Picollo et al., 2016b).

When using KCl as a secretagogue to increase the probability of release from DA neurons, a mean release of 3.7 × 10^4^ DA molecules/spike was found. Comparable neurotransmitter content values (∼3 × 10^4^ DA molecules) were estimated from axonal DA vesicles using CFEs (Sulzer et al., 2016). In other CFE experiments on postnatally derived midbrain neurons, performed at a 100 kHz sampling rate to discriminate between single-spike and flickering events, DA release values were estimated to be around 1 × 10^4^ and 2.4 × 10^4^ DA molecules, respectively for single-spike and flickering events (Staal et al., 2004). It is worth noting that estimates of quantal size can be affected by the different experimental configurations used, such as detection being performed from the cell apex using CFEs or from the cell bottom using μG-SCD-MEAs (Amatore et al., 2007).

### Detection of Spontaneous Firing From Cultured Midbrain Neurons

Besides detecting the quantal release of DA, μG-SCD-MEAs can be exploited to measure the electrical activity of cultured midbrain neurons. The 8 × 8 μG-SCD-MEAs and the conventional MEAs can reveal APs only after 7 DIV in approximately 30% of the electrodes, due to the delayed maturation of the network after dissociation. However, the network activity becomes detectable in most (i.e., 70%) of the electrodes at later stage of maturation (14 DIV). Spontaneous firing at this stage of maturation exhibits great variability of responses, even within the same μG-SCD-MEA or conventional MEA. Neuronal firing frequencies were scattered throughout a range of frequencies varying from 0.1 to 15 Hz (Figure 3C) for both devices, which is in reasonably good agreement with previous reports of low frequency activity in isolated SN DA neurons (Bean, 2007; Margolis et al., 2010). A broad distribution of firing frequencies has also been observed in SN slices positioned onto conventional MEAs: the majority of neurons (∼94%) exhibit low firing frequencies (i.e., 1–3 Hz), whereas the remaining ones fire at higher frequencies (5–10 Hz) (Berretta et al., 2010). Different firing patterns have also been described for *in vivo* recordings, where SN DA neurons display both slow single-spike activity (1–10 Hz), and higher frequency discharges (∼13–20 Hz) (Dragicevic et al., 2015; Hage and Khaliq, 2015). A final consideration concerns the low firing rate (<1 Hz) that was recorded in the majority of neurons, and the hardly detectable DA release that occurred at 0.1 Hz. Both processes suggest the existence of a tonic discharge activity at rest, partially tuned by D_2_-autoreceptors (Al-Hasani et al., 2011).

### Using μG-SCD-MEAs forPharmacological Studies: The L-DOPA-Induced Down-Modulation ofSpontaneous Firing

The neuronal discharge of SN DA neurons is inhibited by D_2_-autoreceptors-mediated GIRK activation and is prevented by the D_2_-antagonist sulpiride (Mercuri et al., 1990; Dragicevic et al., 2014). To assay the sensitivity of the μG-SCD-MEAs, we tested this inhibitory pathway in current-clamped TH-GFP neurons, as well as in mature midbrain DA neurons, cultured for 2 weeks on μG-SCD-MEAs (or conventional MEAs). This autocrine inhibition is induced by adding L-DOPA, which is converted to DA and then released from dopaminergic neurons.

As shown, L-DOPA caused a range of effects (Figure 4). In most current-clamped neurons, the response caused a firing frequency reduction together with a slow membrane hyperpolarization and an increased AP amplitude that was reverted by sulpiride. The increased AP amplitude and the rapid increase in the AP rising phase was revealed as an enhanced *dV/dt* peak amplitude in the phase-plane plot analysis of AP recordings. The same occurrence was detected using both the μG-SCD-MEAs and conventional MEAs (Figure 5). Both MEAs revealed the reduced firing frequency and the increased AP rising phase, which were reverted by sulpiride. It is worth noting that, in the case of MEAs, the increased AP rising phase in current-clamp recordings is converted to an increased peak amplitude in the extracellular APs.

From a physiological point of view, both measurements are in excellent agreement and suggest sustained recruitment of Na_V_ channels due to the increased cell hyperpolarization induced by GIRK K^+^ channel activation. Sustained cell hyperpolarizations increase the rate of Na_V_ channels recruitment from steady-state inactivation (Vandael et al., 2015; Guarina et al., 2018), while the recrutiment of different Na_v_ channel isoform that characterized by a lower threshold of activation cannot be excluded.

Concerning the opposing effect that was observed in a minority of neurons, in which L-DOPA increased the spiking activity (Figure 6), variable responses have also been described in Substantia Nigra pars compacta (SNc) neurons, using MEA recordings from midbrain slices (Berretta et al., 2010). In that case, neurons that fired at high rates (>5 Hz) were insensitive to DA, while low-firing neurons were either highly or weakly inhibited by DA. Furthermore, a fraction of low-rate spiking neurons were insensitive to DA, or excited by DA, and a minority of neurons were potentiated by L-DOPA. Under our experimental conditions, where midbrain neurons were cultured for weeks on the microarray, signals detection may occur from different DA subpopulations (Lammel et al., 2008; Liss and Roeper, 2010), either from non-DA neurons or from DA neurons of the nearby ventral tegmental area. Indeed, the excitatory effects of L-DOPA on nigral dopaminergic neurons have been previously described, and were featured as an “early” and a “late” phase of excitation (Guatteo et al., 2013).

Finally, regarding the fraction of neurons that were inhibited by L-DOPA and did not undergo relevant membrane potential hyperpolarization, several pathways may be responsible for this modulation, based on the involvement of K^+^ channels other than GIRK (Yang et al., 2013), or D_1_-mediated signaling cascades (Surmeier et al., 2007).

## Conclusion

Our data demonstrate that μG-SCD-MEAs are highly reliable as multi-functional sensing multiarrays for long-term recordings of neuronal activity under variable pharmacological conditions. With respect to conventional approaches, the real-time measurements of quantal exocytosis and neuronal firing makes μG-SCD-MEA a promising biosensor for *in vitro* investigation of neuronal circuit properties as well as a valid tool for studying mistuned neurotransmission in neurodegenerative disorders.

## Author Contributions

GT performed the experiments and analyzed acquired data. FP fabricated the sensors, performed the experiments, and manuscript preparation. AB fabricated the sensors. BP contributed to critically editing the manuscript. SDM contributed to critically editing the manuscript. AP made hardware and software of the 16-channel setup and revised the manuscript. PO contributed to the design for the diamond biosensor and to the preparation of the manuscript. AM contributed to experimental design and manuscript preparation. PC contributed to critically editing the manuscript. EC contributed to the interpretation of AP recordings and helped with a critical revision of the manuscript. VC contributed to planning the experimental design, manuscript writing, and overall revision.

## Conflict of Interest Statement

The authors declare that the research was conducted in the absence of any commercial or financial relationships that could be construed as a potential conflict of interest.
